# Potential Use of Halophytes to Remediate Saline Soils

**DOI:** 10.1155/2014/589341

**Published:** 2014-07-06

**Authors:** Mirza Hasanuzzaman, Kamrun Nahar, Md. Mahabub Alam, Prasanta C. Bhowmik, Md. Amzad Hossain, Motior M. Rahman, Majeti Narasimha Vara Prasad, Munir Ozturk, Masayuki Fujita

**Affiliations:** ^1^Department of Agronomy, Faculty of Agriculture, Sher-e-Bangla Agricultural University, Sher-e-Bangla Nagar, Dhaka 1207, Bangladesh; ^2^Laboratory of Plant Stress Responses, Department of Applied Biological Science, Faculty of Agriculture, Kagawa University, 2393 Ikenobe, Miki-cho, Kita-gun, Kagawa 761-0795, Japan; ^3^Department of Agricultural Botany, Faculty of Agriculture, Sher-e-Bangla Agricultural University, Sher-e-Bangla Nagar, Dhaka 1207, Bangladesh; ^4^Department of Plant, Soil, and Insect Sciences, University of Massachusetts, Amherst, MA 01003-7245, USA; ^5^Subtropical Field Science Center, University of the Ryukyus, 1 Senbaru, Nishihara City, Okinawa 903-0213, Japan; ^6^Institute of Biological Sciences, Faculty of Science, University of Malaya, 50603 Kuala Lumpur, Malaysia; ^7^Department of Plant Sciences, University of Hyderabad, Andhra Pradesh 500 046, India; ^8^Department of Biology, Ege University, Bornova, Izmir, Turkey

## Abstract

Salinity is one of the rising problems causing tremendous yield losses in many regions of the world especially in arid and semiarid regions. To maximize crop productivity, these areas should be brought under utilization where there are options for removing salinity or using the salt-tolerant crops. Use of salt-tolerant crops does not remove the salt and hence halophytes that have capacity to accumulate and exclude the salt can be an effective way. Methods for salt removal include agronomic practices or phytoremediation. The first is cost- and labor-intensive and needs some developmental strategies for implication; on the contrary, the phytoremediation by halophyte is more suitable as it can be executed very easily without those problems. Several halophyte species including grasses, shrubs, and trees can remove the salt from different kinds of salt-affected problematic soils through salt excluding, excreting, or accumulating by their morphological, anatomical, physiological adaptation in their organelle level and cellular level. Exploiting halophytes for reducing salinity can be good sources for meeting the basic needs of people in salt-affected areas as well. This review focuses on the special adaptive features of halophytic plants under saline condition and the possible ways to utilize these plants to remediate salinity.

## 1. Introduction

The total area of salt-affected soils in the world is 831 million hectares which includes 397 and 434 million hectares of saline and sodic soils, respectively [1]. The agricultural land is decreasing constantly due to population pressure, adverse environmental condition, continuously increasing natural calamities, and global climate change [2, 3]. More than 45 million hectares of irrigated land are affected by salt which account for 20% of total land and 1.5 million ha of land are taken out of production each year owing to high salinity levels [4, 5]; if it continues in such way, 50% of cultivable lands will be lost by the middle of the 21st century [6].

There are various reasons for salinity including natural (weathering of parent material, deposition of sea salt carried in wind and rain, inundation of coastal land by tidal water, etc.) and anthropogenic activities (rise of water table due to excessive irrigation by underground water, irrigation with salt containing water, poor drainage, etc.) [3, 7, 8]. Increased level of salinity negatively influences germination, plant growth and reproducibility, physiological processes, including photosynthesis, respiration, transpiration, membrane properties, nutrient balance, enzymatic activity, and metabolic activities, cellular homeostasis, and hormone regulation and leads to production of reactive oxygen species (ROS); and in severe stress, it leads to plant death [6, 9]. Salinity is a continuous process and its remediation is cost- and labor-intensive. It is a complex global problem that cannot be solved simply; rather a multidisciplinary approach is required. There are various ways for remediation and proper utilization of saline soils including agronomic practices, use of salt-tolerant crop varieties, and phytoremediation. Among them, phytoremediation can be cost-effective and environmentally sound technology for remediation of salt-impacted sites.

In recent years, plants having ability to remove salts from contaminated soils have been studied and identified by many researchers [10–13]. According to Flowers and Colmer [14], halophytes are characterized as plants that can survive and reproduce in environments where the salt concentration exceeds 200 mM of NaCl (~20 dS m^−1^). These species constitute approximately 1% of the world's flora. Halophytes are plants capable of completing their life cycle under highly saline (NaCl) conditions [15]. Halophytes are also called euhalophytes because they have increased productivity with increasing salt levels and actually grow better under salinity condition than under fresh water conditions [16]. These halophytes possess special morphological and anatomical features as well as physiological processes which are well suited to cope with saline environments. The halophytes can effectively improve the saline soil as they are well adjusted in salt environment because of their diversified adaptation mechanisms including ion compartmentalization, osmotic adjustment, succulence, ion transport and uptake, antioxidant systems, maintenance of redox status, and salt inclusion or excretion [17]. There are diversified species of halophytes suited to grow in different saline regions throughout the world, namely, coastal saline soil, soils of mangrove forests, wet land, marshy land, lands of arid and semiarid regions, and agricultural fields. So, these plants can be grown in land and water containing high salt concentration, can be substitute for conventional crops, and can be good source of food, fuel, fodder, fiber, essential oils, and medicine [17]. At the same time, halophytes can be exploited as significant and major plant species bearing potential capability of desalination and restoration of saline soils and phytoremediation as well. By developing these precious strategies, unused and marginal land can be brought under cultivation and existing agricultural land will be more productive which will open a new door to sustain crop productivity. Considering the above facts, in this review, we have focused on the potential use of halophytes in remediation of salt-affected soils.

## 2. Halophytes

**Figure 1:**
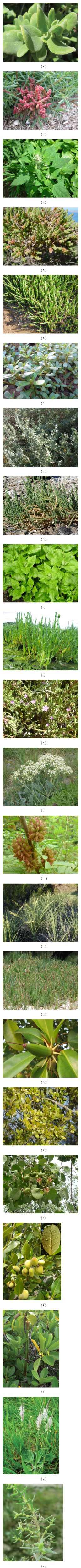
List of major halophytes discussed in this paper. (a)* Mesembryanthemum crystallinum*, (b)* Suaeda australis*, (c)* Chenopodium album*, (d)* Salsola vermiculata*, (e)* Sarcocornia quinqueflora*, (f)* Portulaca oleracea*, (g)* Atriplex* spp., (h)* Allenrolfea occidentalis*, (i)* Tetragonia tetragonioides*, (j)* Salicornia europaea*, (k)* Sesuvium portulacastrum*, (l)* Crambe maritima*, (m)* Glycyrrhiza glabra*, (n)* Distichlis spicata*, (o)* Sporobolus virginicus*, (p)* Bruguiera gymnorrhiza*, (q)* Aegiceras corniculatum*, (r)* Sonneratia apetala*, (s)* Avicennia marina*, (t)* Rhizophora mucronata*, (u)* Plantago media*, and (v)* Suaeda maritima*.

Halophytes are defined in different ways by many scientists based on different criteria. Schimper [18] defined halophytes as the plants capable of normal growth in saline habitats and also able to thrive on “ordinary” soil. According to Stocker [19], they are plants which can tolerate salt concentrations over 0.5% at any stage of life. More simply, Dansereau [20] mentioned that plants which grow exclusively on saline soil are halophytes. Greenway and Munns [21] defined halophytes as follows: “a kind of native flora of saline soils, which contain solutions with a Psi of at least 3.3 bar, being equivalent to 70 mM monovalent salts.” Plants that cannot survive in these habitats are classified as nonhalophytes. Unquestionably, this definition is not quite complete since there is a continuum from the least to the most salt-tolerant species. Some nonhalophytes can also survive in this kind of habitat and complete their life cycle, for example, sugar beet [22]. Some of the major halophytes are listed in Figure 1.

**Figure 2:**
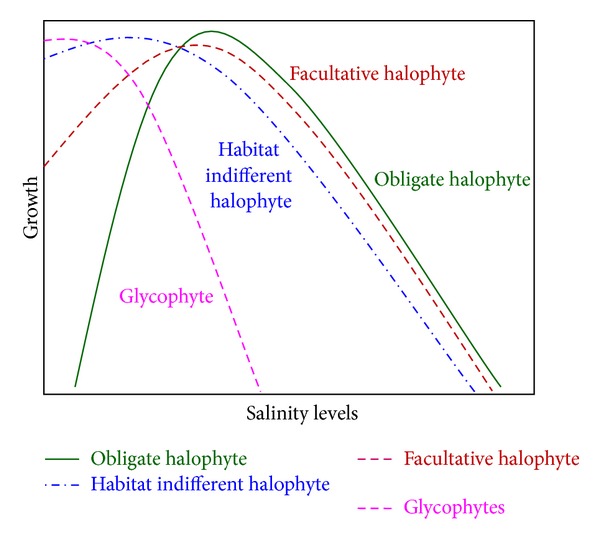
Possible growth pattern of halophyte under saline condition.

Based on ecological aspect, halophytes can be classified as (i) obligate, (ii) facultative, and (iii) habitat-indifferent halophytes [23]. Their growth pattern under saline condition is different (Figure 2). Obligate halophytes grow only in salty habitats. They show sufficient growth and development under high saline condition. Many plant species belonging to Chenopodiceae family fall in this category. Facultative halophytes are able to establish themselves on salty soils, but their optimum lies in a salt-free or at least low-salt condition. However, they can tolerate salt. Most Poaceae, Cyperaceae, and Brassicaceae species as well as a large number of dicotyledons like* Aster tripolium*,* Glaux maritima, Plantago maritima, *and so forth belong to this group. Plants that are indifferent toward their habitat are still able to cope with salty soils in nature. However, they usually grow on salt-free soils. They can compete with species that are sensitive towards salt and are on the other hand able to live on salty soils.* Chenopodium glaucum, Myosurus minimus, *and* Potentilla anserina *can grow in any habitat. In many species, such as* Festuca rubra, Agrostis stolonifera, *and* Juncus bufonius,* the populations living on salty soils and those on salt-free soils differ genetically [23]. However, all of these three kinds of halophytes perform better growth compared to glycophytes (Figure 2).

**Figure 3:**
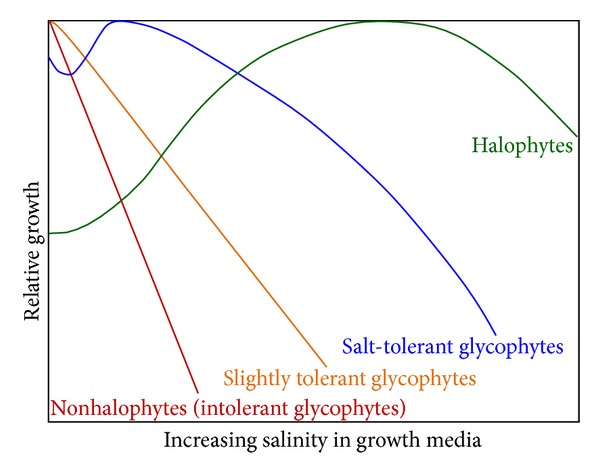
Schematic illustration of growth of different kinds of plants under saline condition.

However, based on growth response curves, Kreeb [24] has coined four plant types (Figure 3). He stated that halophytes are those plants which exhibit maximal relative growth under some salinity in the substrate. Although the growth in halophytes is enhanced by salinity in contrast to nonhalophytes, it is still questionable whether halophytes need salt obligatorily. As obligatory halophytes would only strive successfully on saline soils the question arises if they can live on nonsaline soils [25].

## 3. Mechanism of Adaptation of Halophytes under Saline Condition

**Figure 4:**
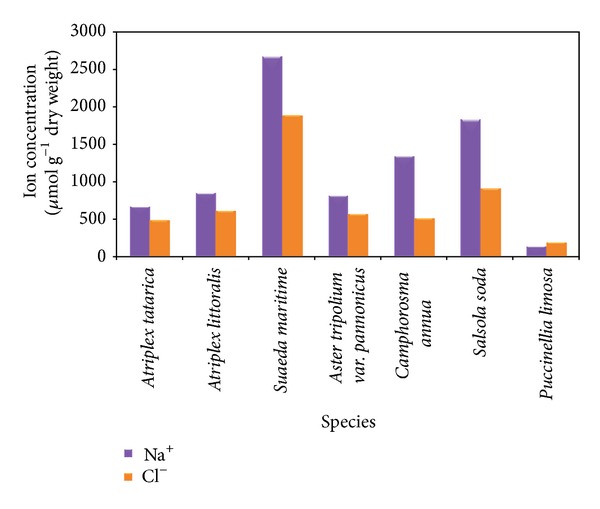
Na^+^ and Cl^−^ ions concentration in the shoot of some halophytes grown in natural habitats [3] with permission from Springer.

Salinity is not inimical to all plants. The distribution, exploitation [26], and physiology of salt tolerance of halophytes are intensively studied [16, 27, 28]. Salts taken up by halophytes do not directly control plant growth by affecting turgor, photosynthesis, or the activity of one or another enzyme. The build-up of salts in old leaves hastens their death. The loss of leaves affects the supply of assimilates or hormones to the growing organs and hereby affects growth [29, 30]. Despite their polyphyletic origins, halophytes appear to have evolved the same basic method of osmotic adjustment: accumulation of inorganic salts, mainly NaCl, in the vacuole and accumulation of organic solutes in the cytoplasm. Differences between halophyte and glycophyte ion transport systems are becoming apparent. The pathways by which Na^+^ and Cl^−^ enter halophyte cells are not well understood but may involve ion channels and pinocytosis in addition to Na^+^ and Cl^−^ transporters. Na^+^ uptake into vacuoles requires Na^+^/H^+^ antiporters in the tonoplast and H^+^ ATPases and perhaps PPIases to provide the proton motive force. Tonoplast antiporters are constitutive in halophytes, whereas they must be activated by NaCl in salt-tolerant glycophytes, and they may be absent from salt-sensitive glycophytes. Halophyte vacuoles may have a modified lipid composition to prevent leakage of Na^+^ back to the cytoplasm [31]. It is also to be noted that halophytes often possess large vacuoles. For example,* Suaeda maritime*, a potential halophyte, occupies 77% of the mesophyll cells of vacuoles [32] which makes it capable of accumulating higher concentration of salt as much as 500 mM [33]. Moreover, Na^+^ concentration of the cell sap even exceeded 800 mM in another halophyte,* S. maritime* [34]. Although all of the halophytes exhibit better accumulation of salt, the level of total salt accumulation in the shoot is mostly species specific, depending on different adaptive strategies ([34]; Figure 4). Based on numerous studies, several adaptative mechanisms were recognized in relation to salt tolerance, which include ion compartmentalisation, osmolyte production, germination responses, osmotic adaptation, succulence, selective transport and uptake of ions, enzyme responses, salt excretion, and genetic control [35].

Based on the different mechanisms of adaptation to salty condition, Walter [36] has classified the halophytes into 3 types: (i) salt excluding, (ii) salt excreting, and (iii) salt accumulating (Table 1).

Since ionic toxicity caused by Na^+^ and Cl^−^ is the main concern of salt stress in plants, most studies have concentrated on Na^+^ exclusion and the control of Na^+^ transport within the plant [5]. Halophytes are able to tolerate high ionic concentration which involves the ability to reduce the ionic stress on the plant by minimizing the amount of Na^+^ that accumulates in the cytosol of cells, particularly those in the transpiring leaves [37]. Although salt exclusion is very efficient way to minimize salt stress, the way of putting off the ions or impairing the uptake is very complex. However, true halophytes are developed with well-developed transport system that can enable a lower uptake and accumulation of salts in the upper parts of the plant, especially in the transpiring organs, especially leaves [38]. Exclusion of Na^+^ happens mainly due to low net Na^+^ uptake by cells in the root cortex and the tight control of net loading of the xylem by parenchyma cells in the stele [39]. Lower permeability root even under excessive concentration of soil salinity also actively helps in salt exclusion [40, 41]. There is plenty of evidence that indicated that Na^+^ exclusion from leaves is associated with salt tolerance in many glycophytes including rice, wheat, and barley [42]. The capacity of salt exclusion is, however, directed by several factors like selectivity of uptake by root cells; preferential loading of K^+^ rather than Na^+^ into the xylem by the cells of the stele; removal of salts from the xylem in the upper parts of roots, the stem, and leaf sheaths, based upon exchange of K^+^ for Na^+^; and loading of the phloem [43]. The capacity of plant to sense Na^+^ is also an important factor which is extracellularly done by a membrane receptor, whereas intracellular Na^+^ may be sensed either by membrane proteins or by any of the many Na^+^-sensitive enzymes in the cytoplasm [37].

**Figure 5:**
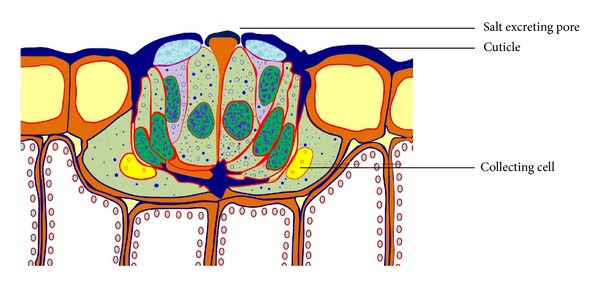
Cross section of a salt gland [3] with permission from Springer.

Among several special characteristics related to the physiological adaptation of halophytes, salt excretion is one of the most efficient mechanisms that prevent excessive concentrations of salts building up in photosynthetic tissues [3]. Some of the halophytes possess multicellular salt glands and salt hairs; those are common in many halophytes such as* Cressa *(Convolvulaceae),* Frankenia *(Frankeniaceae),* Spartina*,* Chloris*, and* Aeluropus *(Poaceae),* Atriplex *(Chenopodiaceae),* Statice*,* Limonium*,* Plumbago*, and* Armeria *(Plumbaginaceae),* Glaux *(Primulaceae),* Tamarix* and* Reamuria *(Tamaricaceae), and some mangrove species, for example,* Avicennia*,* Aegialitis*,* Aegiceras, *and* Acanthus *[3]. These glands are composed of a set of epidermal cells complexes; those capture salt from the mesophyll cells beneath them, to which they are connected by numerous plasmodesmata, and secrete it at the leaf surface, where a layer of salt crystals is formed ([3]; Figure 5). The process of salt excretion by salt gland is yet to be elucidated by some researchers; however, one of the requisites is the availability of energy (ATP) which is required for ion pumping. In halophytes, this energy is provided by the active respiration of the glandular cells [44].

Accumulation of compatible solutes is often regarded as basic strategy for the protection and survival of halophytes under salt stress [45]. These soluble compounds, including soluble carbohydrates, GB, polyols, and Pro [46], protect plants against stress by cellular osmotic adjustment, detoxification of ROS, protection of membrane integrity, and stabilization of enzymes and proteins [47]. Moreover, the leaf tissues of halophytes are adapted to accumulate large amounts of salt ions. Such adaptive mechanism is crucial to generate a water potential gradient along root-shoot to maintain water flux throughout plants [48].

## 4. Potential Use of Halophytes under Saline Condition

Due to the rapid climate change, the saline area in the world is increasing day by day and currently there is an ample need to develop highly salt-tolerant crops to cope with the adverse situation. Halophytes are able to provide satisfactory yield under high salt condition. Some of halophytes producing satisfactory yield under different degrees of salinity are presented in Table 2. There are already several examples known for the utilization of halophytes for industrial, ecological, or agricultural purposes. Halophytes have been tested as vegetable, forage, and oilseed crops in agronomic field trials. The most productive species yield 10 to 20 t ha^−1^of biomass on seawater irrigation, equivalent to conventional crops.* Salicornia bigelovii,* an oilseed halophyte, for example, yields 2 t ha^−1^ of seed containing 28% oil and 31% protein, which is similar to soybean yield and seed quality [31]. Many plant species have been used traditionally as herbs and vegetables and hence rediscovery of the potentials of several promising halophytic plant species to be farmed as leafy vegetables is going on for a couple of decades ([49]; Table 3). Some of the halophytes are good fodder and hence can be used for animal feeding in saline-prone areas. However, it is to be taken into consideration that some halophytes may cause nutritional barrier due to partially high salt content and antinutritional compounds [68].

As the reclamation of salt-affected soils is not completely feasible and is not always cost-effective, the researchers are searching for biosaline agriculture and thus it is obvious to explore a better understanding of how naturally adapted plants (halophytes) handle salts. Study of halophytes can be useful from three perspectives [31]. First, the mechanisms by which halophytes survive and maintain productivity on saline water can be useful to develop tolerant varieties in conventional crops [69–72]. Second, halophytes grown in an agronomic setting can be used to evaluate the overall feasibility of high-salinity agriculture [31, 71]. Third, halophytes may become a direct source of new crops [12, 71, 73–76]. However, halophytes are sometimes unable to perform better in some situations. For instance, halophytes can have low biomass compared to many glycophytes [77].

Apart from accumulation of salt from the saline habitat, many of the halophytes are capable of remediating toxic metals and can grow and give yield. Halophytes are often adapted well in metal-affected habitat compared to glycophytic plants which makes them a good candidate as an ecofriendly and sustainable solution of contaminated coastal environments cleanup [78].

## 5. Phytoremediation

Amelioration of saline and sodic soils has been predominantly achieved through the application of chemical amendments. However, amendment costs have increased prohibitively over the past two decades due to competing demands from industry and reductions in government subsidies for their agricultural use in several developing countries [79]. Since climate and cost are two vital factors in reclamation of saline land, hence, cultivation of salt-tolerant species could be an effective way to improve this situation [80]. Recently, a new environmentally safe and clean technique known as phytoremediation has been introduced to address the salinity problem. This includes the introduction of salt (ion) removing species to control salinity and to maintain the sustainability of agricultural fields [11, 12, 81]. Phytoremediation is defined as the use of plants to remove pollutants from the environment and to render them harmless [82]. These plants not only remediate the salt-contaminated soils but also provide food, fodder, fuelwood, and industrial raw material and increase the income of the farmers owning salt-affected lands. Several halophytic plant species have been tried in the past for their possible use in reclamation of salt-affected soils [81, 83–85]. After conducting number of experiments, several researchers found phytoremediation to be an effective amelioration strategy for calcareous saline-sodic and sodic soils with comparable performance against the use of chemical amendments [86–88]. Besides their positive impact on salt-affected soils, the potential use of some halophytes as forage and as oil seed crops has also been described [31]. According to Qadir et al. [79], phytoremediation has been shown to be advantageous in several aspects: (i) no financial outlay to purchase chemical amendments, (ii) accrued financial or other benefits from crops grown during amelioration, (iii) promotion of soil-aggregate stability and creation of macropores that improve soil hydraulic properties and root proliferation, (iv) greater plant-nutrient availability in soil after phytoremediation, (v) more uniform and greater zone of amelioration in terms of soil depth, and (vi) environmental considerations in terms of carbon sequestration in the postamelioration soil.

In Pakistan, Chaudhri et al. [89] investigated the ability of* Suaeda fruticosa* to accumulate sodium and other salts and reported that the leaves of this plant were found to contain 9.06% salt on a fresh weight basis. Ravindran et al. [81] observed that* S. maritima* and* Sesuvium portulacastrum* exhibited greater accumulation of salts in their tissue and higher reduction of salts from the saline land. It is estimated that these two halophytes could remove 504 and 474 kg of NaCl, respectively, from the saline land from 1 ha in 4-month time. Boyko [90] was the first person to suggest that halophytic plants could be used to desalinate soil and water. The hypothesis set forth by Boyko does not distinguish between sodium and other salts. Zahran and Abdel Wahid [91] made an attempt to reclaim poorly drained soils in Egypt using* Juncus rigidus* and* J. acutus* and reported that the EC of soil had a 50% saturation decreased from 33 to 22 dS m^−1^ in a single growth period. Bioreclamation of saline-sodic soil by Amshot grass (*Echinochloa stagnina*) in Northern Egypt, Helalia et al. [92] reported that, when compared to ponding and gypsum treatment, Amshot grass reduced the exchangeable sodium percent of the surface layer of the soil. Ke-Fu [93] found that* Suaeda salsa* produces about 20 tons dry weight ha^−1^ containing 3-4 tons of salt. Hamidov et al. [94] reported that* Portulaca oleracea* accumulated highest salt uptake (497 kg ha^−1^) with biomass production of 3948 kg ha^−1^. Rabhi et al. [11] reported that* Arthrocnemum indicum*,* Suaeda fruticosa,* and* Sesuvium portulacastrum* seedlings grown on a saline soil significantly reduced the soil salinity and EC by absorbing soluble salts mainly sodium ions and they also reported that* Sesuvium portulacastrum* was able to accumulate nearly 30% of Na^+^ content in shoot over the 170 d. Nasir [95] conducted a field study in Jordan valley to investigate the effects of growing three types of salt accumulator halophyte species,* Tamarix aphylla*,* Atriplex nummularia*, and* A. halimus*, on chemical properties of saline sodic soil and these halophytic species decreased the soil salinity at the end of the experiment. Rabhi et al. [96] observed that* Sesuvium portulacastrum*, an obligate halophyte, decreased the soil salinity and sodicity.

Vegetative bioremediation or bioreclamation of salt-affected soils is an economic solution mainly for developing countries since chemical additions are becoming increasingly expensive. Several authors [13, 81, 91–93, 97–100] have proved that the potential of halophytic plants to accumulate enormous salt quantities depends often on the capacity of their above ground biomass (hyperaccumulating plants). This ability could be of great importance, particularly in arid and semiarid regions, where insufficient precipitations and inappropriate systems [101] are unable to reduce the salt burden in the rhizosphere of plants [102] and suitable physicochemical methods are too expensive. Environmentally safe and clean technique to address the salinity problem includes the introduction of salt (ion) removing species to control salinity and to maintain the sustainability of agricultural fields. Large-scale decontamination of soils and underground water using phytoremediation techniques requires plants with high salt uptake rates, large biomass, and tolerance to a wide array of environmental conditions and constraints. Furthermore, salt marshes, especially salt accumulating halophytes, are dominant crop in the coastal region and introduction of these salt removing halophytic species could potentially create both environmental and economic solutions to remediate saline soils. After reclamation studies are over, these cultivated halophytes can be utilized as animal fodder or for making organic composts.

Akhter et al. [80] evaluated the phytoremediation performance of salt-tolerant species* Leptochloa fusca *(L.) Kunth (kallar grass) in salt-affected soils. They observed that soil salinity, sodicity, and pH decreased exponentially by growing kallar grass as a result of leaching of salts from surface (0–20 cm) to lower depths (>100 cm). Concentrations of soluble cations (Na^+^, K^+^, Ca^2+^, and Mg^2+^) and anions (Cl^−^, SO_4_^2−^, and HCO_3_^−^) were reduced through to greater soil depths. The decline in soil pH was attributed to release of CO_2_ by grass roots and solubilization of CaCO_3_. The ameliorative effects on the soil chemical environment were pronounced after three years of growing kallar grass. Cultivation of kallar grass enhanced leaching and interactions among soil chemical properties and thus restored soil fertility. The soil maintained the improved characteristics with further growth of the grass up to five years suggesting that growing salt-tolerant plants is a sustainable approach to biological amelioration of saline wastelands [80]. Very recently, de Souza et al. [103] reported* Atriplex nummularia* Lindl. as a very potential halophyte that sustains under water-stressed condition on sodic or saline soil. They concluded that the growth patterns and anatomical changes shown by the halophyte* A. nummularia* Lindl. grown under different soil moisture conditions can contribute significantly to the management of soil and water in semiarid regions.

Phytoremediation could become a cost-effective and environmentally sound technology for remediation of salt-impacted sites if it can be properly developed. There are certain limitations that must be overcome for this plant-based remediation system to come into common usage. Phytoremediation can be time-consuming because it requires several growing seasons to lower the level of contaminants in soil. It is also limited to soil depths that are in the rooting zone [104]. Furthermore, successful remediation of soil with high levels of salt is hard to achieve by the fact that plant growth and germination are inhibited by salinity. As a result, finding salt-tolerant plants that have deep and vigorous root growth, as well as sufficient above-ground biomass production, is one of the basic criteria for the selection of plants for remediation of salt-impacted sites. However, selecting suitable plant species for the phytoremediation of salt-affected soils is also important. According to Qadir and Oster [105], plants having capability to remove the maximum quantity of salts by producing higher biomass with some economic importance are mainly selected for phytoremediation [105]. The selected plant species should tolerate high salt concentration. The plants which provide food and fodders are very effective to be used for phytoremediation. Several plant species including grasses, shrubs, and trees are being used for phytoremediation of salinized soils. Many trees have also been recommended. Phytoremediation with trees and grasses is beneficial because these can be utilized as fodder, timber, and fuel [12, 106, 107].

However, use of halophytes for soil reclamation is still in an exploratory stage and only a few field studies for bioreclamation of saline soil using halophytes have been carried out so far and therefore, more research is needed to study the utilization of halophytes to remove excess salinity added by irrigation.

## 6. Conclusion and Future Perspectives

It is clear that salinity problem is increasing rapidly throughout the world. More than half a billion hectares of land are not being properly used for crop production. Thus, there is a need to search for means to improve saline soils so that such soils could support highly productive and meaningful land-use systems to meet the current challenges of global food security. In addition, the crop adaptability to saline conditions should also be improved. Despite the importance of salinity in shaping the composition of coastal plant communities, our knowledge about how different species respond physiologically to variable salinities is limited. In particular, our understanding of physiological/biochemical mechanisms underlying halophytes under variable salinities is very scarce. Hence, the physiological and molecular studies to reveal the underlying mechanisms of these processes are important. In addition, discovering the induction of signaling cascades leading to profound changes in specific gene expression is also considered an important salt stress adaptation. Molecular knowledge of response and tolerance mechanisms will pave the way for engineered plants that can tolerate salt stress and could be the basis for production of crops which can result in economic yield under salt-stress conditions. In recent years, phytoremediation of saline soils has been studied by researchers and it was observed that the use of some halophytes could remove salt from soil. Phytoremediation could become a cost-effective and environmentally sound technology for remediation of salt-impacted sites if it is properly developed. There are certain limitations that must be overcome for this plant-based remediation system to come into common usage. Phytoremediation can be time-consuming because it requires several growing seasons to lower the level of contaminants in soil. It is also limited to soil depths that are in the rooting zone. It is necessary to find the plants having capability to remove the maximum quantity of salts by producing higher biomass with some economic importance are mainly selected for phytoremediation and the selected plant species should tolerate high salt concentration. The forthcoming challenge for using halophytes to remediate soil salinity is to develop a plant with diverse salt accumulating capacity in a cost-effective way. Identification of novel genes with high biomass yield characteristics and the subsequent development of transgenic plants with superior remediation features would be crucial for such type of research.

## Figures and Tables

**Table 1:** Walter's classification of halophytes [36].

Types of halophytes	Characteristics and examples
Salt excluding	In these plants, the root system possesses an ultrafiltration mechanism and this characteristic leads to establishment of such species as the dominant component of the mangrove vegetation. Example: *Rhizophora mucronata*, *Ceriops candolleana*, *Bruguiera gymnorrhiza*, and *Kandelia candel*.

Salt excreting	These plants regulate internal salt levels through foliar glands. Example: *Avicennia officinalis*, *Avicennia alba*, *Avicennia marina*, *Aegiceros corniculatum,* and *Acanthus ilicifolius*.

Salt accumulating	They accumulate high concentrations of salt in their cells and tissues and overcome salt toxicity by developing succulence. Example: *Sonneratia apetala*, *Sonneratia acida*, *Sonneratia alba*, *Limnitzera racemosa*, *Excoecaria agallocha*, *Salvadora persica*, *Sesuvium portulacastrum*, *Suaeda nudiflora,* and *Pentatropis sianshoides*.

**Table 2:** Plant species commonly found in halophytic environments and their salt tolerance limit. Adapted from Ventura and Sagi [49] with permission from Elsevier.

Plant species	Salt tolerance limit	References
*Aster tripolium *	40 mM	Ventura and Sagi [49]
*Atriplex lentiformis *	500 mM	O'Leary et al. [50]
*Atriplex triangularis *	150 mM	Gallagher [51]
*Batis maritima *	500 mM	O'Leary et al. [50]
*Salicornia europaea *	500 mM	O'Leary et al. [50]
*Salicornia persica *	100 mM	Ventura et al. [52]
*Sarcocornia fruticosa *	100 mM	Ventura et al. [52]
*Aster tripolium *	300 mM	Koyro et al. [35]
*Atriplex hortensis *	>250 mM	Wilson et al. [53]
*Batis maritima *	200 mM	Debez et al. [54]
*Cochlearia officinalis *	100 mM	de Vos [55]
*Crambe maritima *	>100 mM	de Vos et al. [56]
*Crithmum maritimum *	150 mM	Hamed et al. [57], Ben Amor et al. [58]
*Diplotaxis tenuifolia *	~150 mM	de Vos [55]
*Inula crithmoides *	400 mM	Tardío et al. [59], Zurayk and Baalbaki [60]
*Mesemyranthenum crystallinum *	400 mM	Herppich et al. [61], Agarie et al. [62]
*Plantago coronopus *	250 mM	Koyro [63]
*Portulaca oleracea *	<140 mM	Simopoulos [64], Yazici et al. [65]
*Salicornia* sp.	>500 mM	Ventura et al. [66]
*Sarcocornia* sp.	>500 mM	Ventura et al. [66]
*Tetragonia tetragonioides *	174 mM	Wilson et al. [53], Słupski et al. [67]

**Table 3:** List of halophytes with highest potential as vegetable crop for saline irrigation. Adapted from Ventura and Sagi [49] with permission from Elsevier.

Plant species	Salt tolerance limit	Popular uses	References
*Aster tripolium *	300 mM	Fresh salads, cooked vegetable	Koyro et al. [35]
*Atriplex hortensis *	>250 mM	Pot herb, colorful salad greens	Wilson et al. [53]
*Batis maritima *	200 mM	Eaten raw, cooked, or pickled	Debez et al. [54]
*Cochlearia officinalis *	100 mM	Fresh salads	de Vos [55]
*Crambe maritima *	>100 mM	Fresh salads	de Vos et al. [56]
*Crithmum maritimum *	150 mM	Fresh and pickled as spice and for salads	Hamed et al. [57], Ben Amor et al. [58]
*Diplotaxis tenuifolia *	~150 mM	Mixed salads	de Vos [55]
*Inula crithmoides *	400 mM	Salads, pickled in vinegar	Tardío et al. [59], Zurayk and Baalbaki [60]
*Mesemyranthenum crystallinum *	400 mM	Salad green or quickly cooked	Herppich et al. [61], Agarie et al. [62]
*Plantago coronopus *	250 mM	Salad greens	Koyro [63]
*Portulaca oleracea *	<140 mM	Salad greens, cooked vegetable	Simopoulos [64], Yazici et al. [65]
*Salicornia *sp.	>500 mM	Salad greens, vegetable	Ventura et al. [66]
*Sarcocornia *sp.	>500 mM	Salad greens, vegetable	Ventura et al. [66]
*Tetragonia tetragonioides *	174 mM	Frozen like spinach	Wilson et al. [53], Słupski et al. [67]
